# MicroRNA-130b is involved in bovine granulosa and cumulus cells function, oocyte maturation and blastocyst formation

**DOI:** 10.1186/s13048-017-0336-1

**Published:** 2017-06-19

**Authors:** Pritam Bala Sinha, Dawit Tesfaye, Franca Rings, Munir Hossien, Michael Hoelker, Eva Held, Christaine Neuhoff, Ernst Tholen, Karl Schellander, Dessie Salilew-Wondim

**Affiliations:** 1 Institute of Animal Science, Department of Animal Breeding and Husbandry, University of Bonn, Endenicher Allee 15, 53115 Bonn, Germany; 2 Teaching and Research Station Frankenforst, Faculty of Agriculture, University of Bonn, Frankenforsterweg 4, 53639 Königswinter, Germany; 3 Center of Integrated Dairy Research, University of Bonn, Meckenheimer Allee 172, 53115 Bonn, Germany; 4 Present address: Department of Biotechnology, Engineering and Applied Sciences, Amity University Ranchi, Ranchi, Jharkhand 834002 India; 5 Present address: Department of Animal Breeding and Genetics, Bangladesh Agricultural University, Mymensingh, -2202 Bangladesh

**Keywords:** Oocyte, Embryo, miR-130b, Mitochondrial activity

## Abstract

**Background:**

Oocyte maturation and preimplantation embryo development are controlled by array of genes that are post-transcriptionally regulated by microRNAs. With respect to this, previously, we identified altered expression of microRNA-130b (miR-130b) during oocyte maturation. Here, we aimed to investigate the role of miR-130b in bovine granulosa and cumulus cell function, oocyte maturation and preimplantation embryo development using gain- and loss-of- function approach.

**Methods:**

For this study, the granulosa cells, cumulus cells and the oocytes were collected from ovaries obtained from slaughterhouse. The genes targeted by miR-130b were identified using dual-luciferase reporter assay. The role of miR-130b in granulosa and cumulus cell function was investigated by increasing and inhibiting its expression in in vitro cultured cells using miR-130b precursor and inhibitor, respectively while the role of miR-130b on oocyte development, immature oocytes were microinjected with miR-130b precursor and inhibitor and the polar body extrusion, the proportion of oocytes reaching to metaphase II stage and the mitochondrial were determined in each oocyte group 22 h after microinjection. Moreover, to investigate the role of miR-130b during preimplantation embryo development, zygote stage embryos were microinjected with miR-130b precursor or inhibitor and the cleavage rate, morula and blastocyst formation was analyzed in embryos derived from each zygote group after in vitro culture.

**Results:**

The luciferase assay showed that SMAD5 and MSK1 genes were identified as the direct targets of miR-130b. Overexpression of miR-130b increased the granulosa and cumulus cell proliferation, while inhibition showed the opposite phenotype. Apart from these, modulation of miR-130b altered the lactate production and cholesterol biosynthesis in cumulus cells. Furthermore, inhibition of miR-130b expression during oocyte in vitro maturation reduced the first polar body extrusion, the proportion of oocytes reaching to metaphase II stage and the mitochondrial activity, while inhibition of miR-130b during preimplantation embryo development significantly reduced morula and blastocyst formation.

**Conclusion:**

This study demonstrated that in vitro functional modulation of miR-130b affected granulosa and cumulus cell proliferation and survival, oocyte maturation, morula and blastocyst formation suggesting that miR-130b is involved in bovine oocyte maturation and preimplantation embryo development.

**Electronic supplementary material:**

The online version of this article (doi:10.1186/s13048-017-0336-1) contains supplementary material, which is available to authorized users.

## Background

Development of oocyte starts in the fetal ovary, while final oocyte growth and maturation occurs during the adulthood [1]. During this long meiotic arrest, the oocyte increases in size by acquiring the maternal transcripts and proteins necessary for embryo development at earlier stages [2]. The growth and development of the oocyte is influenced by the functional activities of the surrounding somatic cells (cumulus or granulosa and theca cells). These cells regulate various kinds of hormones, proteins, metabolites and regulatory molecules via gap junctions, ultimately leading to the development and maturation of the oocyte [3]. These processes are in turn, post-transcriptionally regulated by the activation or disappearance of various cell signaling molecules that are essential for regulating the fate of the cell cycle.

MicroRNAs (miRNAs) are small, non-coding RNA molecules that are involved in the post-transcriptional regulation of gene expression [4–9]. These tinny non coding RNAs are believed to be involved in many functions, such as ovarian function, early embryonic development, granulosa cell proliferation, and stem cell differentiation [10–16]. Moreover, miRNAs are found to be expressed in ovaries [17], mature and immature oocytes, cumulus cells, and preimplantation embryo [18, 19]. Inline to these, identification and functional characterization of the miRNAs in oocyte maturation and preimplantation embryo development have been the focus of research interest. On this regard, Byrne and his colleague [20] showed the involvement of miRNA in the regulation of genes associated with early embryonic development in mice and others reported embryonic lethality in mice due to functional loss of miR-290–295 cluster [21]*.* Moreover*,* the appearance and disappearance of specific sets of miRNAs during embryonic development in various animal species have been described in many instances [18, 19, 22–25].

The role of miRNAs in oogenesis could be inferred from the fact that miRNAs regulate ovarian function, prevent granulosa cell apoptosis, and control hormonal secretion in granulosa cells [26]. In our group, the expression level of miRNAs in bovine immature and maturated oocytes was analyzed using a heterologous approach [18]. From that study, including miR-130b, a total of 59 miRNAs were differentially expressed between the two oocyte groups. Of those, miR-130b was more interesting, as it belongs to the miR-130 family and this miRNA is known to be conserved in vertebrates [12]. In addition, miR-130b is highly expressed in mouse mammary tumor [27], liver cancer [28, 29], mesenchyma stromal cells [30], fibroblast cells [31], gastric cells [32], human mammary epithelial cells [27], and glioma cells [33]. Increased expression of miR-130b was also found to be associated with the proliferation of pancreatic cancer [34]. However, the role of miR-130b in bovine granulosa and cumulus cell development, oocyte maturation, and preimplantation embryonic development is not yet known. Therefore, here, we aimed to examine the role of miR-130b in bovine granulosa and cumulus cell function, oocyte maturation, and preimplantation embryonic development using miRNA gain- and loss-of-function approaches.

## Methods

In this study, the role of miR-130b in bovine granulosa and cumulus cell function, oocyte maturation, and preimplantation embryonic development was investigated using two strategies. In the first phase of the study, the expression profile of miR-130b in granulosa cells, immature oocytes and corresponding cumulus cells, matured oocyte and corresponding cumulus cells and preimplantation embryos was investigated. Following this, the miR-130b target genes were in silico analyzed and validated using the dual luciferase assay. In the second phase of the study, the role of the miR-130b in granulosa and cumulus cell function were investigated by overexpression or inhibition of miR-130b expression by transfecting the cumulus or granulosa cells with miR-130b precursor or inhibitor while the role of miR-130b on oocyte maturation and preimplantation embryo development was investigated by microinjecting the GV stage oocyte and zygote, respectively with miR-130b precursor or inhibitor. The details of the materials and methods used for this study are described bellow.

### Immature oocytes, immature cumulus and granulosa cell collection

Prior to functional analysis, the expression profile of miR-130b was analyzed in granulosa cells, immature oocytes and corresponding cumulus cells, matured oocyte and corresponding cumulus cells and preimplantation embryos. For this, bovine ovaries were obtained from the local slaughterhouse and transported to the laboratory within 2–3 h in a thermo flask (35 °C) containing physiological saline (0.9% NaCl). The follicular contents were then aspirated from 2 to 8 mm diameter of follicular size using 18-gauge needle and collected in 50 ml tubes. The cumulus oocyte complexes (COC’s) were then collected under the stereomicroscope (Nikon) and washed three times in drops of pre-warmed maturation medium. The granulosa cells were obtained by centrifugation of the follicular fluid. The immature COC’s were treated with hyaluronidase 1 mg/ml (Sigma) to separate the immature oocyte and immature cumulus cells. The granulosa cells, immature oocyte (*n* = 100) and cumulus cells obtained from immature oocytes were frozen for genetic analysis.

### Metaphase II (MII) oocyte) and cumulus cell collection

The COCs were obtained as described above and in vitro matured in maturation media at 39 °C in the incubator with humidified atmosphere of 5% CO_2_ in air for 22–24 h using a similar protocol described previously [18]. At the end of maturation, the COCs were treated with 1 mg/ml of hyaluronidase (Sigma) and vortexed for 4 min to separate the oocyte and cumulus cells. The matured oocytes (MII) (*n* = 100) and the corresponding cumulus cells were washed two times in PBS (Sigma) and frozen separately in cryo-tubes containing 20 μl of lysis buffer [0.8% IGEPAL (Sigma), 40 U/μl RNasin (Promega Madison WI, USA) and 5 mM dithiothreitol [(DTT) (Promega Madison WI, USA)].

### In vitro embryo culture

For collection of preimplantation embryo, the mature oocytes were in vitro fertilized and in vitro cultured as described previously [35]. Following this, 2-cell (*n* = 100), 4-cell (*n* = 75), 8-cell (*n* = 50), morula (*n* = 50) and blastocyst (*n* = 50) stage embryo were collected for miR-130b and its target genes expression analysis.

### Total RNA isolation from oocyte granulosa & cumulus cells, oocytes, and preimplantation embryos and complementary DNA synthesis for expression analysis of miR-130b and its target gens

Total RNA containing miRNAs was isolated from three independent pools of immature and in vitro matured oocytes and their corresponding cumulus cells; granulosa cells and preimplantation embryos (zygotes; 2-cell, 4-cell, and 8-cell stage embryos; morula; and blastocysts) using the miRNeasy mini kit (Qiagen, Hilden, Germany). The total RNA from cumulus and granulosa cells was eluted in 30 μl of RNase-free water and, 20 μl RNase-free water from oocyte and embryo samples. The quantity of RNA was determined using NanoDrop 8000 spectrophotometer (NanoDrop, Wilmington, Delaware, USA). Following this, the total RNA was reverse transcribed using a miScript reverse transcription kit (Qiagen, Hilden, Germany) for miRNA analysis and using superscriptase II (Invitrogen), random primer and oligodT_23_ for mRNA analysis.

### Expression analysis of miR-130b in oocyte companion cells, oocytes, and preimplantation embryos using quantitative real time PCR (qPCR)

The expression pattern of miR-130b was analyzed in cDNA samples obtained from granulosa cells, immature and matures oocytes and their corresponding cumulus cells, and preimplantation embryos using quantitative real time PCR (qPCR). For this, mature miRNA-130b specific primers were purchased from Qiagen (Hilden, Germany). The qPCR was performed by mixing 2.5 μl template cDNA with 12.5 μl of SYBRGreen mix (Qiagen, Valencia, CA), 10× miScript Universal Primer and 10× miScript Primer assay in 25 μl of final volume. The qPCR was performed for 45 cycles of 95 °C for 15 s and 60 °C for 1 min in 7000 Real-Time PCR system (Applied Biosystems, USA). The threshold cycle (C_t_) values of miR-130b and the endogenous controls were recorded using Sequence Detection Software (SDS v1.2.1, Applied Biosystems, USA). The qPCR data were normalized using the geometric mean of U6 and small nuclear RNAs (Snord48). The miRNA expression levels were then determined from the triplicate runs using the 2^-ΔΔCt^ method. All experiments were performed at least in three biological triplicates.

### In situ localization of miR-130b in ovarian tissue

In situ localization of miR-130 in ovarian section was performed according to previously described protocols [17, 36]. Briefly, 10 μm sections of the ovarian tissue were mounted on poly-L-lysine coated slides (Menzel GmbH & Co. KG, Braunschweig, Germany) and fixed in 4% (*w*/*v*) paraformaldehyde. After washing with PBS, the sections were incubated in 50%, 70%, 90% and 100% ethanol (*v*/v) followed by a descending alcohol series of 90%, 70%, 50% ethanol (*v*/v) for 5 min each, respectively and then were washed in 1× PBS for 5 min. The samples were then blocked in 0.6% (*v*/v) H_2_O_2_ for 1 h followed by twice washing with 1× PBS for 5 min of each. The samples were then acetylated using 0.1 M TEA buffer and 0.25% acetic anhydride for 10 min and equilibrated in 2× SSC for 10 min. The samples were then incubated with 3′-Digoxigenin (DIG) labeled LNA-modified oligonucleotide probes (1 pM) of miR-130b, U6 or scramble RNAs (Exiqon, Vedbaek, Denmark) in hybridization buffer in a humidified chamber at the temperature 20 °C below the T*m* of probes. After overnight incubation, the samples were washed briefly in wash buffer (similar to hybridization buffer but without tRNA) and serial wash in 2X SSC/wash buffer (each time 10 min) to final three washes in 0.2X SSC each for 30 min at hybridization temperature was performed. Afterwards, blocking, incubation with anti-DIG-AP antibody, washing and color development (Fast Red substrate reaction) was performed. Finally, the samples were then mounted with VectaShield containing DAPI (Vector laboratories, Burlingame, CA) and analyzed by confocal laser scanning microscope (CLSM LSM-510, Carl Zeiss, Germany).

### Whole mount in situ localization of miR-130b in oocytes and preimplantation embryo

A minimum of 5 COCs or preimplantation embryos (zygotes, 2-cell, 4-cell, 8-cell, morula and blastocyst) were used for in situ hybridization. For this, the COCs or embryos were fixed in 4% paraformaldehyde overnight and transferred to 100% methanol for rehydration and acetylation. Following this, the samples were treated with 10 μg/ml Proteinase K for 10 min and then washed in PBS for 10 min. Two hours of pre-hybridization was performed at 52 °C in hybridization solution (50% formamide, 5× SSC, 0.1% Tween-20, 50 μg/ml heparin, and 500 mg/ml yeast tRNA). Embryos were incubated overnight with 3′-Digoxigenin (DIG) labeled LNA-modified oligonucleotide probes (1 pM) for mir-130b, together with scrambled RNAs (Exiqon, Vedbaek, Denmark) in hybridization buffer in a humidified chamber at the temperature 20 °C below the T*m* of probes. After overnight incubation, the embryos were washed briefly in wash buffer (similar to hybridization buffer but without tRNA) and serial wash in 2XSSC/wash buffer (each time 10 min) to final three washes in 0.2X SSC each for 30 min at hybridization temperature was performed. Blocking, incubation with anti-DIG-AP antibody, washing and color development (Fast Red substrate reaction) was performed as described previously [36]. Embryos were mounted individually with VectaShield containing DAPI (Vector laboratories, Burlingame, CA) and images were acquired by a confocal laser scanning microscope (Carl Zeiss, Germany) using Z-stacks with a 1-μm interval (when needed). Three-dimensional images from all the layers or Z positions of single oocytes, follicles, or embryos were constructed using ZEN 2008 Light Edition software (Carl Zeiss, Germany).

### Granulosa and cumulus cell culture

Granulosa and cumulus cells were cultured using a standard cell culture protocol [37, 38] with slight modifications. Briefly, bovine ovaries were collected from local abattoirs and transported to the laboratory in a thermo flask containing 0.9% saline solution at 37 °C. Granulosa cells were harvested from follicular fluid after centrifugation and cumulus cells were collected after denudation of the cumulus oocyte complex (COCs) from 2 to 8 mm diameter of follicular size. All cells were washed 3 times and resuspended in Dulbecco Modified Eagle Medium (DMEM) containing penicillin/streptomycin (200 U/mL) and fungizone (100 mg/mL). Cells were resuspended in DMEM with sodium bicarbonate (10 mmol/l), sodium selenite (4 ng/ml), bovine serum albumin (BSA), (0.1%; Sigma-Aldrich), penicillin (100 U/mL), streptomycin (100 mg/mL), transferrin (2.5 mg/mL), and essential and nonessential amino acids. The viability of freshly harvested cells was estimated using 0.4% trypanblue stain (Sigma) and 8 × 10^4^-2 × 10^5^ cells were cultured in 24-wells plate for 24–48 h depending on the objective of the experiment.

### Validation of genes targeted miR-130b

Prior to performing target gene validation using dual luciferase assay, the potential genes targeted by miR-130b were predicted using PicTar (http://www.pictar.org), MIRANDA (http://www.microrna.org/microrna/home.do) and TargetScan (http://www.targetscan.org). Following this, eight potential target genes (SMAD5, RPS6KA5 (MSK1), MEOX2, MARCH2, DDX6, EIF2C1, EIF2C4, and DOC1R) with strong thermodynamic values were selected. Afterwards, 200–500 base pairs flanking the miR-130b binding site and Pme 1 and Xhol restriction sites in the 3′ UTR of the target genes and a gene sequence (mismatch) that is not target by miR-130b were amplified by polymerase chain reaction (PCR) using gene-specific primers (Additional file 1: Table S1). The mismatch sequence was used as a control. The PCR products were then cloned to the pmirGLO Dual-Luciferase miRNA target reporter vector (Promega). The sequence specificity and orientation were further confirmed by sequencing using CEQTM 8000 Genetic Analysis sequencer (Beckman Coulter, Krefeld, Germany). Afterwards, sub confluent cumulus cells were co-transfected with 800 ng/ml of pmirGLO Dual-Luciferase miRNA target reporter vector harboring of the 3′ UTR of the target genes or a mismatch of the target gene and 50 pmole/ml of miR-130b precursor, inhibitor or mismatch control using Lipofectamine 2000 transfection reagent (Invitrogen) in Opti-MEM medium I reduced serum Media. The miRNA-130b precursors, which mimic endogenous miRNA-130b, miRNA-130b inhibitors that specifically bind to the endogenous miR-130b and scramble miRNA which don’t target any annotated genes were purchased from Ambion (USA). Transfected cells were then collected 48 h post-transfection and the cell lysates were prepared in 100 μl of 1× passive lysis buffer (Promega). The luminescence activity of each sample was then measured with Dual-Luciferase® Reporter Assay System in an Opticom II luminometer (Bretford Instruments).

### Expression analysis of genes targeted by miR-130b in oocyte companion cells, oocytes, and preimplantation embryos

The expression level of the miR-130b target genes in granulosa cells, immature cumulus, mature cumulus, and all preimplantation stage embryos or different treatment groups were determined using the relative standard curve method according to Larionov et al. [39] and the manual described by Applied Biosystems (http://tools.thermofisher.com/content/sfs/brochures/cms_042380.pdf] The standard curve for each gene was generated using the serial dilution prepared from plasmid DNA that consisting the PCR product of each gene. For this, sequence-specific primers (Additional file 2: Table S2) were designed using Primer Express® Software v2.0 (Applied Biosystems, Foster City, CA, USA) and Primer3 (http://frodo.wi.mit.edu/primer3/). All primers were purchased from Eurofins MWG synthesis GmbH (MWG Biotech, Eberberg, Germany). Using the primers, PCR was performed in 20-μl reactions (final volume) containing 2 μl of 10× PCR buffer (Sigma), 0.5 μl of each primer (10 pmole), 0.5 μl of dNTP (50 μM), 0.5 U of Taq DNA polymerase (Sigma), and 14.4 μl of Millipore H_2_O, which was finally added to 2 μl of cDNA. The PCR reactions were run as follows: denaturation at 95 °C for 5 min, followed by 35 cycles at 95 °C for 30 s, annealing at the corresponding temperature for 30 s, and extension at 72 °C for 1 min, with a final extension step at 72 °C for 10 min. The PCR product was then purified using the QIAquick PCR purification kit (Qiagen) and ligated to the pGEM-T Vector System (Promega) and transformed into competent cells. Following this, bacteria with the DNA insert of interest were screened by the presence of β-galactosidase activity and further cultured. After overnight culture, the plasmid DNA was isolated from the bacteria by using a GenEluteTM Plasmid Miniprep Kit (Sigma, Germany). The concentrations of the plasmids were measured using NanoDrop 8000 spectrophotometer (NanoDrop, Wilmington, Delaware, USA). The serial dilution (10^1^–10^9^ molecules) was prepared from each plasmid by converting the concentration of plasmid (ng/μl) into number of molecules using the program that converts the weight (weight concentration) in to molar quantity (molar concentration) and vice versa (http://molbiol.ru/eng/scripts/01_07.html). The geometric mean of the glyceraldehyde 3-phosphate dehydrogenase (GAPDH) and histone (H_2_A) expression levels was used for normalization.

### Cell viability and cell proliferation assay

To understand the role of miR-130b on oocyte companion cells, 7.5 × 10^4^ cells/ml of granulosa or cumulus cells were seeded and sub-confluent cells were transfected with 50 nM of miR-130b precursor, inhibitor, or mismatched using Lipofectamine 2000 reagent (Invitrogen). Following this, the granulosa and cumulus cell viability and proliferation were determined using the modified 3-(4, 5-dimethyl-2-thiazolyl)-2, 5-diphenyl-2H-tetrazolium, bromide (MTT) assay (Sigma) and direct cell staining with trypanblue solution 24 and 48 h post-transfection. The live cell count was done using hemocytometer after trypanblue staining while the MTT activity was determined by measuring the optical density (OD) at 570 nm on a multi-well spectrophotometer (Bio-Rad, model 450, Hercules, CA, USA). All readings were taken within 30 min to 1 h and the mean OD values were determined in each treatment group.

### Cholesterol assay in cultured cumulus cells

To investigate the effects of miR-130b on the cholesterol synthesis activity of oocyte companion cells, about 2 × 10^5^ cumulus cells were cultured in 24-well plates. Sub-confluent cells were then transfected with miR-130b inhibitor, precursor, or scramble sequence. Twenty-four hours post-transfection, the cumulus cells and spent medium were collected and the cholesterol assay was performed using an EnzyChrom™ AF Cholesterol Assay Kit (BioAssay Systems) following the manufacturer’s protocol. Briefly, cumulus cells from each treatment group were resuspended in 20 μl methanol + −chloroform solution. The samples were then centrifuged and the supernatant was transferred into a new tube. The samples were then air-dried and resuspended in 80 μl of assay buffer. Prior to analysis, a 10-fold diluted 100 mg/dl standard was prepared by mixing 15 μl of 300 mg/dl Standard and 435 μl of Assay Buffer, and 50 μl of diluted standards were transferred into wells marked as standard and then 50 μl of 10-fold diluted samples (10 μl sample with 90 μl Assay Buffer) was transferred into sample in 96-wells plate. Following this, 50 μl of the mix (1 μl enzyme, 1 μl Dye Reagent, and 55 μl assay buffer) were added to each well and then the plate was incubated at room temperature in the dark for 30 min. The fluorescence reading was then performed at l_ex_ = 530 nm and l_em_ = 585 nm using the following formula: Cholesterol (mg/dl) = [FSample – Fblank]/Slope, F = fluorescence.

### Glycolysis assay in cultured cumulus cells

To determine whether miR-130b is involved in glycolysis, cumulus cells were transfected with 50 nM of miR-130b precursor, inhibitor, or mismatch in 24-wells plate in serum-free medium. Twenty-four hours post-transfection, the concentration of lactate, the end product of glycolysis, was determined using the lactate colorimetric assay kit (Abcam, Cambridge, MA, USA). The OD was measured at 450 nm and the standard curve plot (nmol/well vs. OD 450 nm) was then generated. Finally, the lactate concentrations were determined as follows: C = La/Sv (nmol/μl or mM), where La is the lactic acid amount (nmol) and Sv is the sample volume (μl) in the well.

### Microinjection of oocytes and in vitro maturation

The ovaries were collected from a nearby slaughterhouse and transported to the IVF laboratory in a thermo flask containing 0.9% saline solution at 37 °C. Cumulus-oocyte complexes (COCs) were then aspirated from follicles with 2- to 8 mm in diameter. Only high quality oocytes (based on their morphological characteristics, mainly the intactness of the cumulus cells and cytoplasmic appearance) were selected for microinjection, and cumulus cells were partially removed to avoid any technical difficulties during microinjection. Prior to microinjection, immature oocytes were incubated for 20 min in TCM-199 supplemented with cytochalasin B, at a final concentration of 8 mg/ml, in order to reduce mechanical damage during injection [40]. The oocytes were then injected with 10 pl of 50 nM miR-130b precursor, miR-130b inhibitor, or mismatch. The oocytes were then subjected to in vitro maturation in an incubator at 38.7 °C and 5% CO_2_ in humidified air. Afterwards, the maturation rate and mitochondrial activity of each group of oocytes were determined. The expression levels of miR-130b and its target genes were also analyzed in the MII-stage oocytes derived from each injected oocyte group.

### Mitochondrial assay in metaphase II (MII) oocytes

The mitochondrial assay was performed in oocytes 22 h post miR-130b precursor, inhibitor, or scrambled sequence injection. For this, in vitro-matured oocytes of different groups were washed in maturation medium and then incubated with 300 nM of mitochondrion-specific dye (MitoTracker® Deep Red, Invitrogen) for 10 min. The oocytes were then washed three times in maturation medium and fixed with 4% paraformaldehyde for 30 min [41]. Following this, the oocytes were washed three times in PBS and the image showing the signal intensity of mitochondrial activity was captured using confocal laser scanning microscope (Carl Zeiss, Germany) at 579 nm to 599 nm wavelengths using Z-stacks with a 1-μm interval.

### Microinjection of zygotes and in vitro culture

Good quality oocytes retrieved from ovaries collected from the slaughterhouse were in vitro matured and fertilized using frozen semen (1×10^6^ spermatozoa/ml) in a fertilization medium that consisted of Fert-TALP medium supplemented with 10 mM sodium lactate, 1 mM sodium pyruvate, 6 mg/ml BSA, 1 μg/ml heparin, 10 μM hypotaurine, 20 μM penicillamine, and 2 μM epinephrine at 38.7 °C and 5% CO_2_ in humidified air. Twenty-two hours after, the cumulus cells were removed and cumulus-free presumptive zygotes were microinjected with 10 pl/zygote of 50 nM miR-130b precursor, inhibitor, or scrambled sequence. Zygote groups injected with scrambled sequence and uninjected groups were used as controls. The zygotes were then in vitro cultured in 400 μl of culture medium in four-well dishes (Nunc, Roskilde, Denmark) covered with mineral oil at 38.7 °C in 5% CO_2_ in humidified air. Following this, the proportions of zygotes that were cleaved and developed to morula or blastocysts were recorded. The expression levels of miR-130b and its target genes were also analyzed in blastocysts derived from each zygote group.

### Protein isolation and western blot

Protein for western blot analysis was extracted from the oocytes and embryo samples used for total RNA extraction using the miRNeasy mini kit (Qiagen, Hilden, Germany). After the centrifugation step described above, 750 μl of isopropanol were added to the organic phase. After 10 min, the samples were centrifuged at full speed for 10 min. The pellets were then washed in 0.3 M guanidine hydrochloride and centrifuged at 7500 x g for 5 min. Finally, the protein pellets were air-dried and dissolved in 200 μl of radioimmunoprecipitation assay buffer (RIPA) buffer (Sigma) containing 1% protease inhibitor cocktail (Sigma) and stored at −20 °C for western blot analysis.

To isolate protein from cultured cells, the cells were washed two times with PBS without calcium and magnesium. Following this, 100 μl of RIPA buffer (Sigma) containing 1% protease inhibitor cocktail (Sigma) were added to the cell lysate and incubated on ice. The lysate was centrifuged and the protein solution was transferred into the new tube and stored at −20 °C for western blot analysis.

Protein analysis of genes targeted by miR-130b was performed in oocyte companion cells, oocytes, and the embryos of different treatment groups using the western blotting technique. For this, proteins were separated by 10% SDS-PAGE and transferred to nitrocellulose membranes (Amersham Pharmacia, Freiburg, Germany). Membranes were then blocked in 1× Roti-Block (Roth GmbH) with 1× Tris-Buffered Saline containing 0.1% Tween-20 (TBST). Afterwards, the membranes were incubated with rabbit polyclonal anti-RPS6KA5 (1:1000) (Acris Antibodies GmbH), goat polyclonal anti-SMAD1/5 (1:500), and anti-GAPDH (1:500) primary antibodies at 4 °C overnight. At the end of the incubation, the membranes were washed 6 times in TBST and incubated with a horseradish-peroxidase (HRP) conjugated donkey anti-rabbit secondary antibody (Santa Cruz Biotechnology) or HRP-conjugated donkey anti-goat antibody (Santa Cruz Biotechnology) at a 1:20,000 dilution factor in 0.1× Roti-Block buffer. The membranes were finally washed in TBST. The protein and antibody binding was detected using the SuperSignal West Pico Chemiluminescent Substrate (Thermo Scientific) and visualized using BioRad (Germany).

### Data analysis

In this study, the relative abundance of miRNA expression between treatment groups was performed using the comparative C_t_ method and the statistical analysis was performed on the fold changes after normalizing the C_t_ values using the geometric mean of U6 and SNORD. The gene expression (mRNA) was determined using standard curve method and normalization was performed using the geometric mean of GAPDH and H2A. One way analysis of variance (ANOVA) followed by Tukey post-hoc multiple pair-wise comparison was performed to test the significant difference of the normalized fold change values of the miRNA or the normalized mean values of the mRNA between treatment groups using GraphPad Prism version 5 (GraphPad software Inc.). Similarly, differences in the cell viability, glycolysis, lactate production, oocyte polar body extrusion of the oocytes, the cleavage rates. Morula and blastocyst rates of the embryos between the treatment groups were also analyzed using one way ANOVA followed by multiple pair-wise comparisons using the Tukey multiple comparison test. Mean differences with *p* ≤ 0.05 were considered to be significantly different.

## Results

### The expression patterns of miR-130b in oocytes, oocyte companion cells and preimplantation stage embryos

**Fig. 1:**
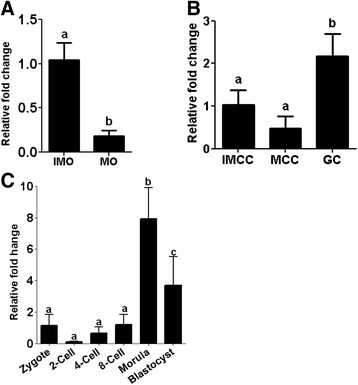
The expression patterns of miR-130b in (**a**) mature (MO) and immature (IMO) oocytes, (**b**) immature (IMCC) cumulus, mature (MCC) cumulus and granulosa (GC) cells, (**c**) preimplantation stage embryos. The expression level of IMO was used as a reference sample to calculate the fold changes between IMO and MO, whereas the expression level of IMCC was used as a reference sample to calculate the fold changes between IMCC, MCC and GC. The expression level of zygotes was used as a reference sample to calculate the fold change expression of miR-130b between preimplantation stage embryos. Bars with different letters are statistically significant (*p* < 0.05). The bars graphs indicate the mean ± standard deviation (SD) from three independent biological samples of MO, IMO IMCC, MCC, zygotes; 2-cell, 4-cell, and 8-cell stage embryos; morula; and blastocysts

The expression pattern of miR-130b was analyzed in immature and in vitro-matured oocytes, cumulus cells and granulosa cells. The result showed that the abundance of miR-130b was significantly higher in the immature oocytes compared to mature oocytes (Fig. 1a). In oocyte companion cells, the expression of miR-130b was relatively higher in granulosa cells (GC) compared to the cumulus cells surrounded the immature (IMCC) or cumulus cells surrounded the mature oocytes (MCC) (Fig. 1b). In addition, the expression pattern of the miR-130b was analyzed during bovine preimplantation embryo stages and result showed that miR-130b was expressed in all stages of preimplantation embryos. The expression level was not significantly different between the embryonic stages before the major embryonic genomic activation. However, the expression level of miR-130b was progressively increased in the morula and blastocyst embryonic stages (Fig. 1c) suggesting that miR-130b could be involved in morula and blastocyst formation.

### In situ localization of miR-130b in follicular cells and preimplantation embryos

**Fig. 2:**
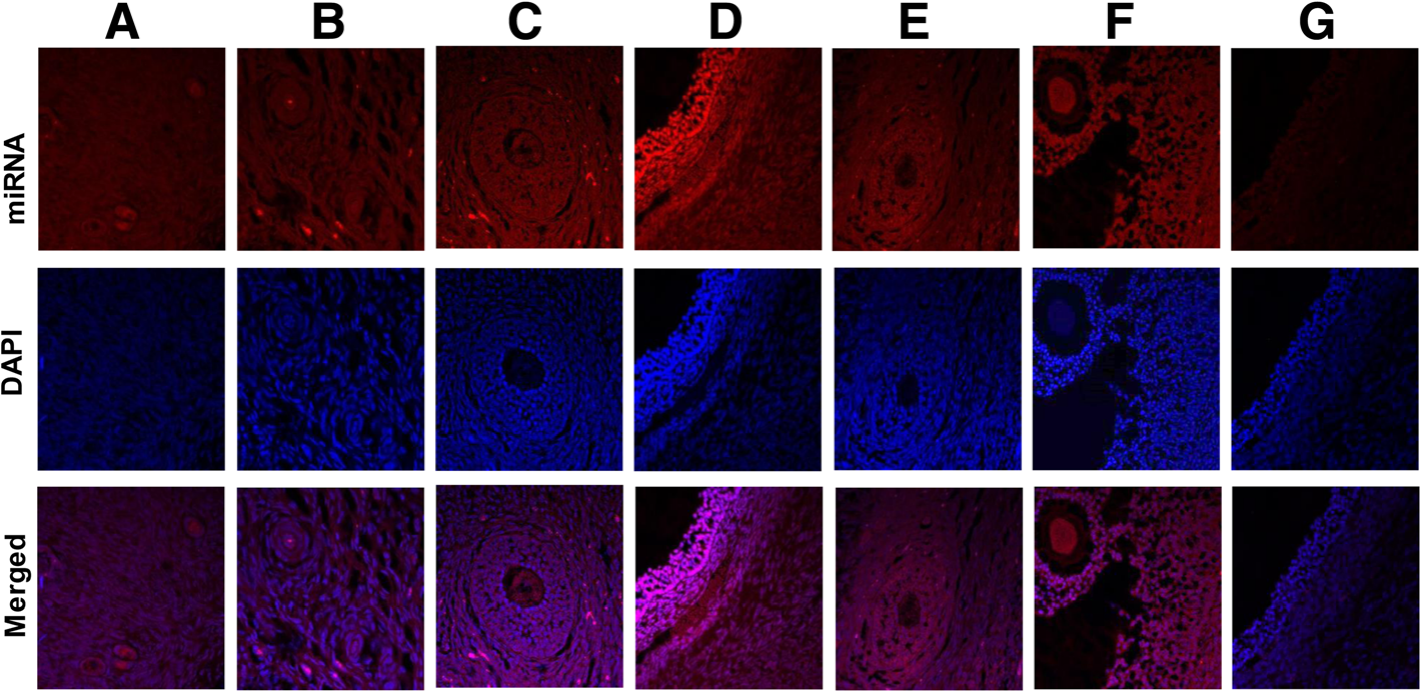
In situ localization of miR-130b in ovarian sections using 3′-digoxigenin labeled locked nucleic acid (LNA) microRNA probe. The signal intensity of miR-130b in primordial (**a**), primary follicle (**b**), secondary follicle (**c**) and antral follicles (**d**). The signal intensity of the U6 miRNA (positive control) is indicated in lanes (**e**) (antral follicle) and (**f**) (secondary follicle); whereas the signal intensity of the scramble miRNA probe (negative control) is indicated in panel (**g**) (antral follicle). The red color indicates the expression level of miR-130b, U6 or scramble miRNA probe while the blue color indicates the nuclear staining using 4′,6-diamidino-2-phenylindole (DAPI)

**Fig. 3:**
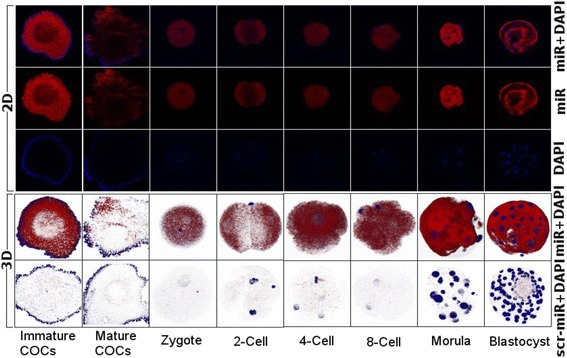
Whole-mount in situ detection of miR-130b expression in oocytes (immature COCs, mature COCs) and preimplantation embryos. The red color indicates the expression level of miR-130b or scramble miRNA probe, while the blue color indicates nuclear staining using 4′,6-diamidino-2-phenylindole (DAPI). The 2D and 3D indicate the two and three dimension images, respectively. COCs; cumulus oocyte complexes

In addition to expression profiling, in situ localization of miR-130b was performed in pre-antral and antral follicles and preimplantation embryos. Accordingly, the signal intensity of miR-130b tended to be stronger in oocyte surrounding cells compared to oocytes in antral follicles, while in preantral follicles, the expression level tended to be similar in oocytes and their surrounding cells (Fig. 2). Similarly, the whole mount in situ localization indicated that lmiR-130b was detected in all preimplantation stage embryos and its expression showed an increasing trend as the developmental stage of the embryo was advancing (Fig. 3).

### MicroRNA-130b targets the SMAD5 and MSK1 genes

**Fig. 4:**
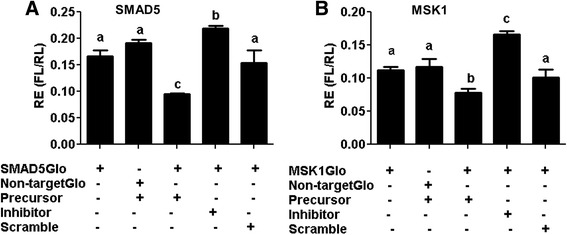
Experimental validation of the target genes of the miR-130b using dual luciferase assay. **a** The luciferase reporter assay in cumulus cells co-transfected with miR-130b precursor or inhibitor and the pmirGLO vector construct harboring of the 3′ UTRs of the SMAD5 gene (SMAD5GLO) or non-target sequence (Non-targetGLO). **b** The luciferase reporter assay in cumulus cells co-transfected with miR-130b precursor or inhibitor and the pmirGLO vector construct harboring of the 3′ UTRs of the MSK1 gene (MSK1GLO) or non-target sequence (Non-targetGLO). The firefly and renilla activity ratio in cells transfected only with vector construct harboring the 3′ UTR of the miR-130b target genes or cells co-transfected along with the miRNA scramble sequence or the firefly and renilla activity ratio of cells co-transfected with miR-130b precursor or inhibitor along with the vector construct harboring the non-target sequence of miR-130b were used as controls. The assay was performed 24 h post transfection. Bars bars with different letters are statistically significant (*p* < 0.05). RE; relative expression; FL; firefly luminescent; RL; renilla luminescent. Bars graphs represent the mean ± standard deviation (SD) and the experiment was repeated three times in three independent samples

To identify the target genes of miR-130b, in silico predicted target genes (SMAD5, RPS6KA5 (MSK1), MEOX2, DOC1R, MARCH2, DDX6, EIF2C1 and EIF2C4) were experimentally validated using dual luciferase assay. The 3′-UTRs of the selected genes and the non-target sequences were integrated into the pmiRGLO vector and co-transfected along with miR-130b precursor, miR-130b inhibitor, or a scrambled miRNA sequence into the cultured cumulus cells. The result showed that the luciferase activity was significantly reduced in the cumulus cells co-transfected with miR-130b precursor and the pmiRGLO vector harboring of the SMAD5 3′-UTR (SMAD5GlO). On the other hand, the luciferase activity was increased in the cumulus cells co-transfected with miR-130b inhibitor and SMAD5GlO compared to cells co-transfected with scrambled miRNA and SMAD5GlO or miR-130b precursor and pmiRGLO harboring the 3′ UTR sequence of the non target sequence (Non-targetGLO) or the cumulus cells transfected only with SMAD5Glo (Fig. 4a).

**Fig. 5:**
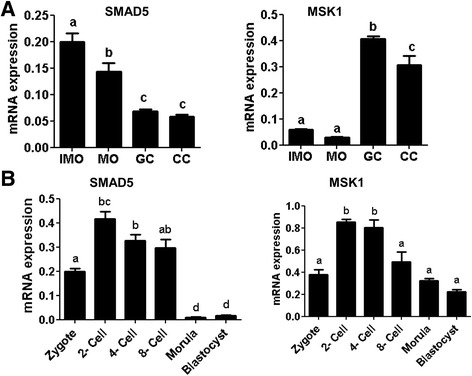
The mRNA abundance level of SMAD5 and MSK1 genes in (**a**) immature oocytes (IMO), mature oocytes (MO), immature cumulus (IMCC), mature cumulus cells (MCC), granulosa cells (GC) (**a**), and preimplantation stage embryos (**b**). Bars with different letters are statistically significant (*p* < 0.05). The bars graphs represent the mean ± standard deviation (SD) and the data was analyzed from three independent biological samples of MO, IMO IMCC, MCC, zygotes; 2-cell, 4-cell, and 8-cell stage embryos; morula; and blastocysts

A similar analysis was performed for the MSK1 gene to confirm if MSK1 is also targeted by miR-130b. Accordingly, the cumulus cells co-transfected with miR-130b precursor and the pmiRGLO vector harboring the MSK1 3′-UTR (MSK1GLO) exhibited a significant decrease in luciferase activity, compared to cells co-transfected with miR-130b precursor and pmiRGLO vector harboring of the mismatch sequence (Non-targetGLO), or cumulus cells co-transfected with scramble miRNA and MSK1GlO, or cells transfected with MSK1GlO alone (Fig. 4b). In addition, EIF2C1, DDX6, DOC1R, and MEOX2 genes were also targeted by miR-130b, although the evidence was not as strong as that of the MSK1 and SMAD5 genes (Additional file 3: Fig. S1). Therefore, SMAD5 and MSK1 were selected as the validated target genes of miR-130b for further functional analysis. Thus, we sought to understand the expression patterns of the SMAD5 and MSK1 genes in granulosa and cumulus cells, oocytes and preimplantation embryos prior to functional analysis. The results showed that the mRNA expression level of SMAD5 was significantly higher in the oocyte compared to its corresponding cumulus cells, whereas the reverse was true for MSK1 gene (Fig. 5a). In preimplantation embryos, the expression of SMAD5 and MSK1 was higher in the 2-, 4- and 8-cell stage embryos, but the expression SMAD5 was significantly reduced in morula and blastocyst stage embryos. (Fig. 5b).

### MicroRNA-130b promotes the viability and proliferation of cumulus and granulosa cells

**Fig. 6:**
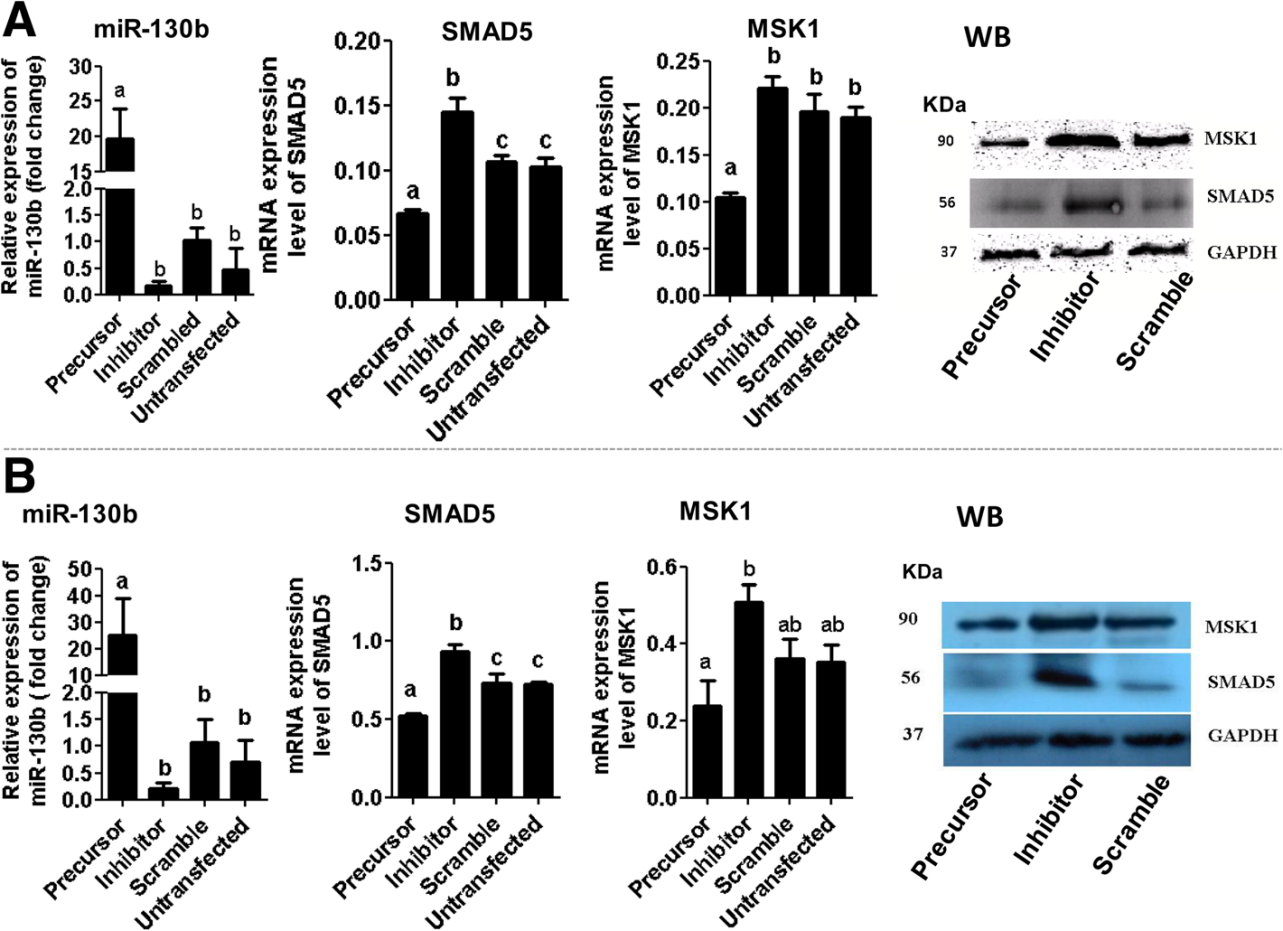
The expression levels of miR-130b and its target genes in cumulus (**a**) and granulosa (**b**) cells transfected with 130b precursor, miR-130 inhibitor and scramble miRNA 24 h post transfection. Granulosa or cumulus cells transfected with scramble miRNA were used as a reference sample to calculate the fold change level of the miR-130b between the treatments. Bars with different letters are statistically significant (*p* < 0.05). The bars graphs indicate the mean ± standard deviation (SD) and the data was analyzed from three independent granulosa and cumulus cell cultures collected from ovaries obtained from slaughterhouse at different days. The GAPDH protein expression level was used to monitor the stability of the housekeeping gene between treatment groups. WB, western blot, KDa; kilo Dalton

To understand the role of miR-130b in oocyte companion cells during oocyte maturation, granulosa and cumulus cells were transfected with miR-130b precursor, miR-130b inhibitor, or miRNA scramble sequence. Twenty-four hours after transfection, the expression level of miR-130b was increased in precursor transfected cumulus (Fig. 6a) and granulosa (Fig. 6b) cells compared to scrambled transfected and untransfected cell groups. Nevertheless, the expression level of miR-130b was not altered in inhibitor transfected group as miRNA inhibitors are not necessary to reduce the expression pattern of the miRNA rather, synthetic miRNA inhibitor are designs to be bind the endogenous mature miRNAs to sequester the endogenous miRNA making it unavailable for normal function. Thus, the efficiency of the miRNA inhibitors is evaluated based on the expression levels of the target genes of that specific miRNA. Therefore, in this study, the expression analysis of genes targeted by miR-130b was analyzed in each cell culture group. The results indicated that the expression level of the SMAD5 gene was decreased in precursor transfected and increased in inhibitor transfected cumulus (Fig. 6a) and granulosa cells (Fig. 6b). Similarly, the expression level of MSK1 tended to be reduced in miR-130b precursor transfected and increased in inhibitor transfected cumulus (Fig. 6a) and granulosa cells (Fig. 6b).

**Fig. 7:**
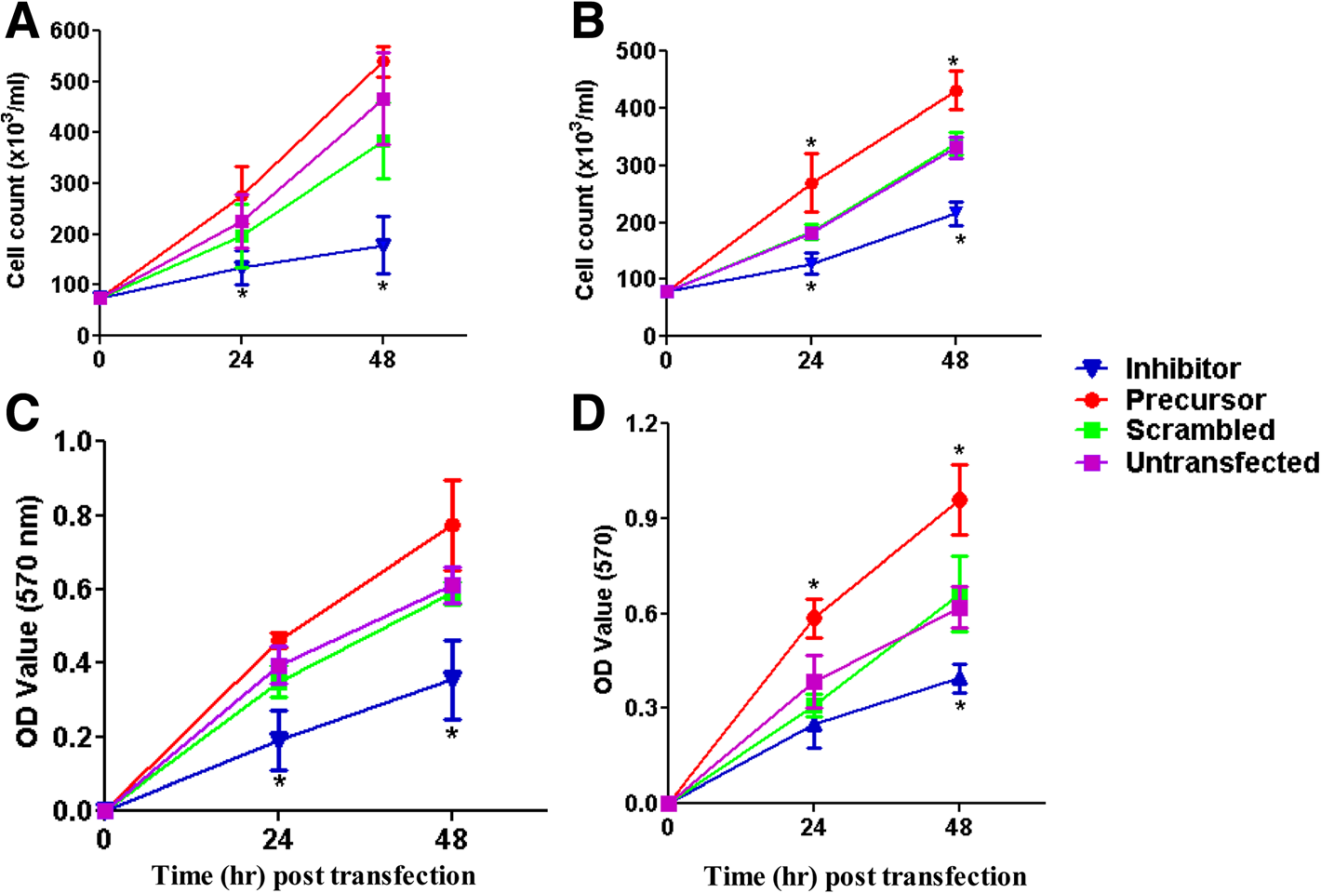
The effect of miR-130b overexpression or inhibition on cumulus and granulosa cell survival and proliferation. The total number of live cumulus (**a**) and granulosa (**b**) cells 24 and 48 h post miR-130b precursor, miR-130b inhibitor, scramble miRNA transfection or untransfected cell groups. The initial plating density (7.5 × 10^4^ cells/ml) was the same in each treatment group before transfection. Direct cell count was performed using trypanblue. The proliferation rate of cumulus (**c**) and granulosa (**d**) cells post miR-130b precursor, miR-130b inhibitor, scramble miRNA transfection and untransfected cell groups determined using MTT assay. Results represent the mean ± standard deviation (SD) of three independent replicates. Significant differences (*p* < 0.05) are indicated by star (*) symbol

Once, we realized that miR-130b and its target genes were altered in cumulus and granulosa cells transfected with miR-130b precursor or inhibitor, the effect of overexpression or inhibition of miR-130b on granulosa and cumulus cells survival and viability have been examined 24 and 48 h post transfection. Since, the initial plating density was similar between the treatment groups; direct cells count was performed at 24 and 48 h post transfection using trypanblue to evaluate the total cell increment in each treatment group. The result showed that, 24 and 48 h post transfection, the total live cells were significantly increased in cumulus and granulosa cells transfected with miR-130b precursor and decreased in cells transfected with miR-130b inhibitor compared to the control groups (Fig. 7a & b). Moreover, the cumulus and granulosa cell proliferation was also determined by colorimetric assay using the 3-[4, 5-dimethylthiazol-2-yl]-2, 5- diphenyltetrazolium bromide; thiazolyl blue (MTT) solution at 570 nm wavelength. The assay results indicated that while overexpression of miR-130b increased, inhibition of miR-130b reduced the granulosa and cumulus cell proliferation compared to scramble and untransfected cell groups (Fig. 7c & d).

### MicroRNA-130b regulates glucose metabolism in cumulus cell

**Fig. 8:**
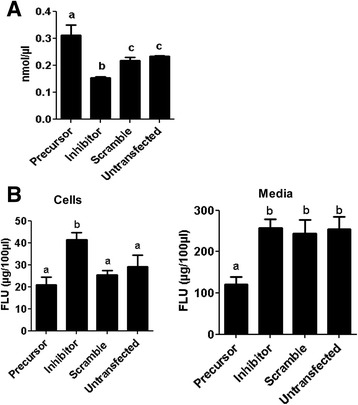
The effect of miR-130b overexpression or inhibition on cumulus cell glycolytic activity (**a**) and cholesterol biosynthesis (**b**). The concentration of lactate which is the end product of glycolysis was determined in the cells, while the cholesterol concentration was determined both in the cells and culture media. RFU: Relative fluorescence units. Bars (mean ± standard deviation) with different letters are statistically significant (*p* < 0.05). The analysis was performed from three independent in vitro cumulus cell culture runs and the cumulus cells were obtained from the cumulus oocyte complexes (COCs) collected from ovaries obtained from slaughterhouse in different days

Since modulation of miR-130b altered the rate of cumulus cell proliferation, we hypothesized that overexpression or inhibition of miR-130b could affect the cumulus cell function by compromising the energy metabolism. Therefore, to determine the role of miR-130b in the energy metabolic activity of the oocyte companion cells, cumulus cells were transfected with miR-130b precursors or inhibitor. The amount of lactate produced by each cell group was then measured to be used as an indicator of glucose metabolism activity of the cells. Accordingly, the lactate production was significantly increased in cumulus cells transfected with miR-130b precursor, and reduced in cells transfected with miR-130b inhibitor compared to scramble transfected and untransfected cell groups (Fig. 8a).

### MicroRNA-130b regulates cholesterol biosynthesis in cumulus cells

Steroidogenesis is one of the major functions of oocyte companion cells during follicular development, and cumulus cells are believed to use high-density lipoprotein cholesterol for steroidogenesis [42]. Therefore, to investigate whether miR-130b is involved in cholesterol biosynthesis in cumulus cells, cholesterol assay was performed in cumulus cells by modulating the expression level of miR-130b. The assay was performed both in the cells and the spent media. The results showed that the cholesterol level was significantly increased in the miR-130b inhibitor transfected compared to scramble transfected and untransfected cell groups. However overexpression of miR-130b didn’t affect the amount of cholesterol synthesis by the cumulus cells (Fig. 8b), but significantly reduced the secretion of the cholesterol to the culture media (Fig. 8b).

### MicroRNA-130b is involved in oocyte maturation

**Fig. 9:**
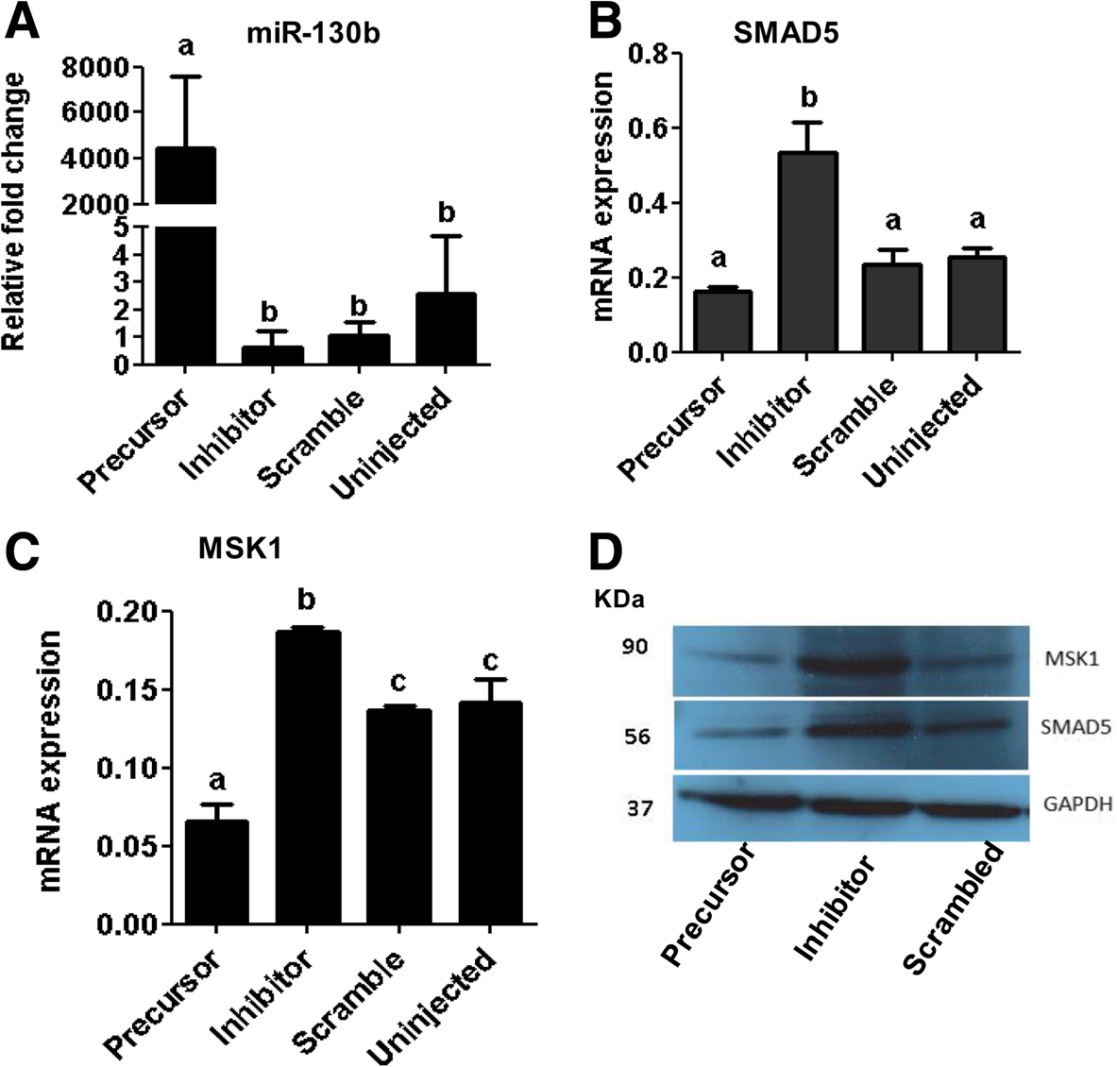
The expression level of miR-130b (**a**), SMAD5 (**b**) and MSK1(**c**) in oocytes derived from GV oocytes injected with miR-130b precursor, miR-130b inhibitor and scrambled miRNA 24 h post injection. The protein expression level of MSK1 and SMAD5 in miR-130b precursor, inhibitor and scrambled miRNA injected oocytes 24 h post injection (**d**). Bars [mean ± standard deviation (SD)] with different letters are statistically significant (*p* < 0.05). The analysis was performed from oocytes obtained from three independent in vitro maturation runs

Since miR-130b was found to regulate the cumulus cells survival and proliferation, cumulus cell glucose metabolic activity and cholesterol production; we further sought to understand whether miR-130b is involved in oocyte maturation. For this, the expression levels miR-130b was modulated during in vitro oocyte maturation by microinjecting miR-130b precursor or inhibitor into the germinal vesicle (GV) stage oocytes. The phenotypic data showed that the first polar body extrusion in oocytes injected with miR-130b inhibitor was significantly lower and tended to be higher in precursor injected group, compared to the oocytes injected with scramble RNA (Table 1). In addition, assessment of the nuclear maturation status of the oocytes using Hoechst-33,342 indicated that significantly higher proportion of oocytes were arrested at telephase-1 and significantly lower proportion of oocytes were reached the MII stage in the miR-130b inhibitor injected oocyte group (Table 2). On the other hand, the proportion of oocytes that reached to the MII stage tended to be higher in precursor injected oocytes, although the difference was not statistically significant compared to uninjected and scramble injected oocyte groups. Following this, the expression profiles of miR-130b and its target genes in MII oocytes derived from different treatment groups were analyzed. The result indicated that the expression level of miR-130b was significantly increased (Fig. 9a), whereas the expression levels (both the mRNA and protein) of SMAD5 (Fig. 9b & d) and MSK1 (Fig. 9c & d) were reduced in MII oocytes derived from GV oocytes injected with miR-130b precursor. The opposite phenomenon was observed in MII oocytes derived from miR-130b inhibitor injected oocytes.

**Table 1:** Polar body extrusion (mean ± SD) in GV oocytes injected with miR-130b precursor, miR-130b inhibitor or scramble miRNA and uninjected groups

Treatment groups	No. of oocytes	First polar body extrusion (%)
miR-130b precursor	356	86.6 ± 3.7^a^
miR-130b inhibitor	489	72.8 ± 5.9^b^
Scrambled	488	83.9 ± 3.5^a, c^
Uninjected	396	80.1 ± 4.0^c^

Different letters of superscripts in the same column indicate significant difference (*P* ≤ 0.05) between treatment groups. *GV* Germinal vesicle, *SD* standard deviation

**Table 2:** The proportion of oocytes (mean ± SD) at GV-arrest, metaphase l or telophase l stages in GV oocytes injected with miR-130b precursor, inhibitor or scramble miRNA and uninjected groups

Treatment groups	No of GV oocytes	GV-arrest	Metaphase l	Telophase l	Metaphase ll
miR-130b precursor	189	8.2 ± 0.1	8.3 ± 3.3	10.1 ± 5.5^a^	73.5 ± 10.1^a^
miR-130b inhibitor	204	8.9 ± 3.1	6.7 ± 3.9	30.1 ± 7.8^b^	52.8 ± 4.4^b^
Scramble	192	7.4 ± 0.9	6.8 ± 4.1	15.3 ± 7.8^a^	69.7 ± 9.7^a^
Uninjected control	191	7.3 ± 0.8	6.7 ± 2.8	13.0 ± 5.9^a^	72.3 ± 6.1^a^

Different letters of superscripts in the same column indicate significant difference (*P* ≤ 0.05) between treatment groups. *GV* Germinal vesicle, *SD* standard deviation

### MicroRNA-130b regulates mitochondrial activity in oocytes during in vitro maturation

**Fig. 10:**
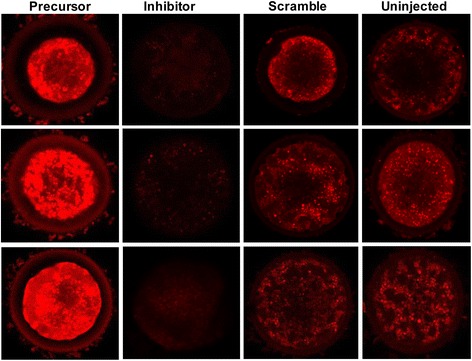
The mitochondrial activity in MII oocytes derived from GV oocytes injected with miR-130b precursor, miR-130b inhibitor or scrambled miRNA

Once we realized that inhibition of miR-130b was significantly reduced the proportion of oocytes that reached the MII stage, the effect of miR-130b on the quality of MII oocytes was investigated by determining the mitochondrial activity. For this, the MII stage oocytes obtained from GV oocytes injected with miR-130b precursor, inhibitor, or scramble miRNA sequence were subjected for mitochondrial assay using mitochondrion-specific dye. At least 20 oocytes per group were taken for microscopy and the experiment was done in triplicate. The results showed that the mitochondrial signal intensity was lower and higher in the MII oocytes derived from miR-130b inhibitor and precursor injected GV oocytes, respectively compared to scramble miRNA injected and uninjected control oocyte groups (Fig. 10).

### Inhibition of miR-130b expression reduced the morula and blastocyst formation

**Fig. 11:**
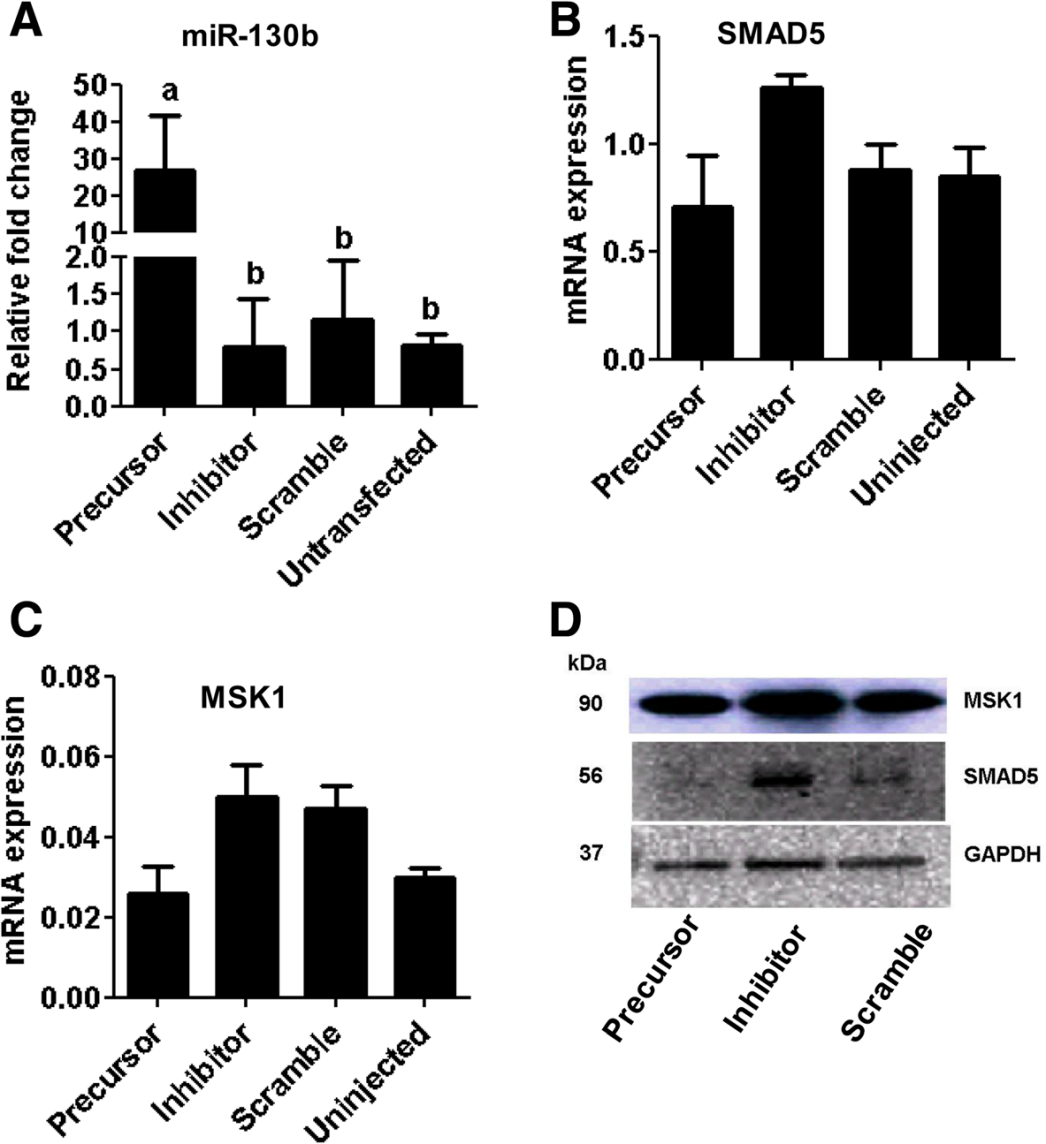
The expression levels of miR-130b and its target genes in blastocysts of different groups. The transcript levels of miR-130b (**a**), SMAD5 (**b**) and MSK1 (**c**) in blastocysts derived from zygotes injected with miR-130b precursor, miR-130b inhibitor, scrambled miRNA or uninjected group. Bars [mean ± standard deviation (SD)] with different letters are statistically significant (*p* < 0.05). The analysis was performed from blastocysts obtained from three independent in vitro cultures. Western blot analysis showing the protein expression level of SMAD5 and MSK1 in blastocyst derived from zygotes of different treatment groups (**d**)

Once we comprehended that miR-130b is involved in oocyte maturation, the role of miR-130b was investigated during the bovine preimplantation embryonic development. For this, first the expression pattern of miR-130b was analyzed in different preimplantation stage embryos. Accordingly, the expression level of miR-130b was not significantly altered from zygote until 8-cell stages. However, significantly higher expression of miR-130b was detected in the morula and blastocyst embryonic stages. Following this, the potential role of miR-130b in preimplantation embryo development was investigated by inhibiting and overexpressing its expression by microinjecting the miR-130b inhibitor and miR-130b precursor in the zygote stage embryos, respectively. Microinjection of miR-130 precursor or inhibitor indicated that although zygotes of different treatment groups showed no significant differences in the cleavage rates 48 h post fertilization, morula and blastocyst formation were significantly reduced in zygotes injected with miR-130b inhibitor, compared to the scramble injected and uninjected control groups (Table 3). Moreover, the blastocyst rate tended to be higher in miR-130b precursor injected zygote group although the difference is not statically significant compared to the scramble injected and uninjected zygote groups. Following this, the expression level of miR-130b and its target genes was analyzed in blastocysts derived from the different zygote groups. The expression miR-130b was significantly increased in the blastocysts derived from miR-130b precursor injected zygotes (Fig. 11a) and the mRNA and protein expression of SMAD5 and MSK1 tended to be higher and lower in blastocysts derived from zygotes injected with inhibitor and precursor, respectively compared to blastocysts derived from zygotes injected with scramble or uninjected groups (Fig. 11b–d).

**Table 3:** The cleavage, morula and blastocyst rates (mean ± SD) in zygotes injected with miR-130b precursor, miR-130b inhibitor or scramble miRNA and uninjected group

Treatment groups	No. zygotes	First cleavage 48 hpi (%)	Morula rate (%)	Blastocyst rate (%)
miR-130b precursor	490	77.1 ± 5.1	32.7 ± 3.8^a^	28.1 ± 2.2^a^
miR-130b inhibitor	451	76.1 ± 0.9	23.2 ± 2.5^b^	21.3 ± 1.9^b^
Scramble miRNA	454	79.9 ± 1.2	27.3 ± 3.8^ab^	25.9 ± 3.1^a^
Uninjected control	326	79.4 ± 5.1	32.7 ± 2.4^a^	27.2 ± 2.6^a^

Different letters of superscripts in the same column indicate significant difference (*P* ≤ 0.05) between treatment groups. *Hpi* hours post insemination (fertilization), *SD* standard deviation

## Discussion

Oocyte maturation occurs by successive transformation of dominant primordial follicle into primary, secondary, and antral follicle. This physiological process is generally governed by induction or degradation of molecules that could be directly or indirectly associated with the cell cycle progression. For instance, altered expression of genes involved in cell-to-cell communication, cell death, cell adhesion, and phosphorylation during oocyte maturation have been documented [43]. Likewise, the miRNAs are also evidenced to show a differential expression pattern during oocyte development and maturation. In this regard, previously, we have identified specific miRNAs whose expression is degraded, induced, or stably expressed during the bovine oocyte maturation [18]. From that specific study, miR-130b was among the several miRNAs whose abundance level was altered during in vitro oocyte maturation. Indeed, in current study, both the qPCR and in situ hybridization indicated that in addition to the oocytes, miR-130b is expressed in bovine oocyte surrounding somatic cells and preimplantation embryos.

Based on the results obtained using qPCR and in situ localization, it was suggested that miR-130b could be involved in regulating the granulosa and cumulus cell function, oocyte maturation and preimplantation embryo development. To confirm this, gain-and loss-of miR-130b function was performed in cumulus and granulosa cells using its precursor and inhibitor as suggested previously [44]. For this, first MSK1 (RPS6KA5) and SMAD5 were identified as the target genes of miR-130b by integrating in silico and wet-lab analyses. The expression analysis of these genes indicated that, the mRNA level of SMAD5 was higher in immature and mature oocytes compared to granulosa and cumulus cells while the expression of MSK1 higher in granulosa and cumulus cells compared to both immature and mature oocytes. Nevertheless, the expression patterns of SMAD5 and MSK1 was quite similar in preimplantation stage embryos where the mRNA levels of both genes were abundant in 2-, 4- and 8-cell stage embryos followed by a decline at the morula and blastocyst stages. Although no report is available regarding the expression pattern of MSK1 in bovine oocyte and preimplantation stage embryos, the expression pattern of SMAD5 was found to be consistent with the previous report [43].

In the current study, overexpression miR-130b promoted granulosa and cumulus cell viability and proliferation; while inhibition of miR-130b resulted in the opposite phenotype. Similar to our findings, overexpression of miR-130b was found to increase the proliferation rates of the esophageal squamous cell carcinoma and U251 cells while suppression of its expression reduced the proliferation rate of both cell types [45, 46]. Furthermore, down-regulation of miR-130b significantly suppressed cell proliferation and induce apoptosis in HL-60 cells [47] suggesting the potential role of miR-130b in cell survival and proliferation. Moreover, in the current study, precursor induced overexpression of miR-130b inhibited the expression (both mRNA and protein) level of MSK1 and SMAD5 gene whereas inhibition of miR-130b increased the protein expression levels of these genes in both cell types. This may indicate that miR-130b could be involved in cumulus and granulosa cell proliferation by modulating the expression patterns of SMAD5 and MSK1 genes. SMAD5 belongs to the SMAD1/5/8 signaling pathways which is believed to promote proliferation and expression of set of genes associated with granulosa cell differentiation by the action of pro-cumulin and cumulin [48]. However, the negative correlation between the SMAD5 gene expression and the proliferative activity of the cells has been described in many occasions. For instance, functional loss or depletion of *SMAD*5 expression promoted ovarian granulosa cell proliferation [49], increased proliferative potential of l colony-forming cells [50], reversed the inhibitory effects of TGF-β on primitive human hematopoietic progenitor cell [51]. Moreover, knockdown of SMAD5 by overexpressing the miR-155 expression enhanced the aggressiveness of diffuse large B cell lymphoma in vivo [52]. On the other hand**,** increased expression of SMAD5 was found to reduce proliferation of cerebellar granule neuron progenitors [53],and induce apoptosis which may have a negative impact on the proliferative activity of the cells [54]. As far as MSK1 is concerned, inhibition of MSK1 resulted in a marked increase in cell production during mouse pancreatic development [55].

Cell proliferation, which is literally defined as increasing in cell number, requires higher energy metabolism during the synthesis and division phases [56, 57]. In many of the proliferating cells, the majority of glucose is converted to lactate and only a small portion is oxidized to carbon dioxide [57]. In line to this, our results clearly showed that while overexpression of miR-130b increased, inhibition reduced the lactate production suggesting that miR-130b is involving in maintaining cumulus cell survival and proliferation by regulating the glycolysis activity. Similar to the current study, the role of miRNAs in glycolytic activity have been described in many instances. For instance, increased level of miR-210 using its mimic increased glycolytic activity in HCT116 cells [58] and downregulation of miR-143 reduced glucose metabolism and inhibits cell proliferation [59].

After comprehending the role of miR-130b in oocyte companion cells survival and proliferation, its role in oocyte maturation was investigated using microinjection strategy. In the current study, inhibition of miR-130b during oocyte maturation significantly affected the oocyte maturation rate, but overexpression had only minimal effect. The lesser effect of overexpression of miR-130b on oocyte maturation could be associated with its abundance level of which its expression was stronger in the GV oocytes compared to the MII ones. Therefore, further overexpressing miR-130b may not have a significant effect on oocyte maturation. However, inhibition of the already accumulated miR-130b using its inhibitor was able to show substantial effect on oocyte maturation. This was also reflected in oocyte companion cells where the inhibition of miR-130b reduced the proliferation rate and the glucose metabolism activity. Thus, it is speculated that miR-130b could affect the oocyte maturation by regulating the proliferation and metabolic activity of the surrounding cells gene. Similarly, in previous study, it was demonstrated that over or under expression of miRNAs in cumulus cells could influence oocyte maturation by affecting the oocyte-cumulus interaction [60]. The signal transduction pathways regulated by miR-130b during the oocyte maturation is not clear: However, when we look into the expression of pattern of the target genes, the expression level of both SMAD5 and MSK1 were significantly increased in oocytes with lower maturation rate suggesting that miR-130b could be involved in oocyte maturation by fine-tuning the SMAD5 and MSK1 genes. Indeed, SMAD5 is one of the key mediators of SMAD dependent transforming growth factor-beta (TGF-beta) superfamily whereas MSK1 involves in the mitogen-activated protein kinase (MAPK) pathway and cross-signaling mechanisms between the two pathways is essential to decide cell fate [61]. In fact, the role of TGF-beta and MAPK pathways in oocyte development has been described in many instances [62–64]. Oocyte maturation is believed to be completed when the oocytes undergo nuclear maturation, cytoplasmic maturation and mitochondria organization. Particularly, the mitochondria activity can be used as indicators of oocyte quality [60, 65, 66]. In this regard, our data demonstrated that modulation of miR-130b during in vitro oocyte maturation significantly affected the mitochondrial activity in the MII oocytes indicating the possible role of miR-130b in regulating the mitochondrial activity during in vitro bovine oocyte maturation. Previously, it has been also suggested that the pattern of the mitochondrial distribution could be the indicators of meiotic progression, cumulus expansion and the oxidative activity during oocytes maturation [67]. Inline to this, previous study has also indicated that reduction in mitochondrial membrane potential in mice embryos lacking functional SMAD5 [27]. Thus, dysfunction of the mitochondrial activity in the oocytes could, in turn, result in reduced blastocyst formation and embryo loss [68].

After investigating its role in oocyte companion cells’ function and oocyte maturation, the role of miR-130b in preimplantation embryo development was examined. The expression of miR-130b was relatively constant until embryonic stages that coincide with bovine major genome activation and then the expression was increased in morula and blastocyst stages of embryos. Interestingly, this was quite opposite to the abundance level of its target genes of which the expression level of both MSK1 and SMAD5 was lower at the morula and blastocysts compared to other preimplantation stages. This may suggest that miR-130b could be embryonic miRNA whereas its target genes are maternal transcripts. Thus, to verify whether miR-130b is involving in preimplantation embryo development, bovine zygotes were injected with miR-130b precursor or inhibitor. Indeed, overexpression or inhibition of miR-130b didn’t affect the cleavage rate of the zygotes 24 h post injection. However, inhibition of miR-130b during preimplantation embryo development affected the morula and blastocysts formation rates indicating the possible involvement of miR-130b in bovine preimplantation development. Similar study in zebra fish also indicated that suppression of endogenous let-7 miRNA was associated with retarded embryo development, a lack of proper eye development, and a reduced tail with yolk sac extension [69]. In addition, the expression analysis of the miR-130b target genes in blastocysts derived from zygotes injected with inhibitor indicated that both the SMAD5 and MSK1 genes were tended to be altered in inhibitor and precursor injected zygote group suggesting that miR-130b could be involving in morula and blastocyst formation by regulating the expression level of SMAD5 and MSK1.

## Conclusion

Here we have shown that in vitro functional modulation of miR-130b using its mimic and inhibitor affected the granulosa and cumulus cell function, oocyte maturation and preimplantation embryo development by targeting SMAD5 and MSK1 genes suggesting that miR-130b is involved in bovine oocyte and preimplantation embryo development. However, a better understanding of miR-130b using a stable knockdown or knock-in experiments could be required to get an in-depth insight about the role of miR-130b in the later stage of embryos development, particularly during the period of embryo implantation.

## Additional files

Additional file 1: Table S1. List of primers used for validation of the miR-130b target genes. (DOCX 15 kb)

Additional file 2: Table S2. List of primers used for quantification of the mRNA abundance level using qPCR. (DOCX 16 kb)

Additional file 3: Figure. S1. The luciferase activity in cumulus cells co-transfected with and miR-130b precursor, miR-130b inhibitor, or scramble sequence with pmirGLO vector construct harboring the 3′ UTRs of EIF2C1, DDX2, EIF2C4, MEOX2 and DOC1R. (TIFF 1411 kb)

## Abbreviations

BSA: Bovine serum albumin
COCs: Cumulus oocyte
DAPI: Complexes 4′,6-diamidino-2-phenylindole
DMEM: Dulbecco Modified Eagle Medium
DTT: Dithiothreitol
FITC: Fluorescein isothiocyanate
GC: Granulosa cells
GV: Germinal vesicle
IMCC: Cumulus cells scrounging immature oocytes
MCC: Cumulus cells surrounding the mature oocytes
miR-130b: MicroRNA-130b
MTT: 3-(4, 5-dimethyl-2-thiazolyl)-2, 5-diphenyl-2H-tetrazolium, bromide
PBS: Phosphate buffer saline
qPCR: Quantitative real time PCR
SSC: Saline Sodium Citrate
