# Supplementary material for: MicroRNA-130b is involved in bovine granulosa and cumulus cells function, oocyte maturation and blastocyst formation
Source: J Ovarian Res. 2017 Jun 19;10:37. doi: 10.1186/s13048-017-0336-1 (PMC5477299; doi:10.1186/s13048-017-0336-1)
Supplement: Supplementary file 1 — List of primers used for validation of the miR-130b target genes. (DOCX 15 kb) [file 13048_2017_336_MOESM1_ESM.docx]

| Gene symbol |  | Primer for 3’UTR 5´ to 3´ (underline bases shows restriction site) | Length  (bp) | Annealing temperature °C) |
| --- | --- | --- | --- | --- |
| SMAD5 | F | GTGCGGTTTAAACGCTAGTGACAGTGCGTGCAT | 194 | Touchdown |
|  | R | GTCGGCCTCGAGAGGGGTACCAAGGAAGCAAG |  |  |
| MSK1 | F | CGCTGTTTAAACAGTTTTGCACTGCTCTTTCC | 483 | 56 |
|  | R | CGTCTCGAGTTGAGCTATACAAGTGCTCTGC |  |  |
| DDX6 | F | CGCTGTTTAAACCTGTGACACATCGATTTTGG | 249 | 58 |
|  | R | GCTCTCGAGAGGCACTTCGCACAAATAAG |  |  |
| EIF2C4 | F | CGCTGTTTAAACGCAACTCGGAATAGTTGCAC | 238 | 58 |
|  | R | CGTGCTCGAGAATTGCCTGTCTGAATCTGC |  |  |
| EIF2C1 | F | CGCTGTTTAAACGCAGAACTGCAACCTTTTGT | 206 | 57 |
|  | R | CGCTCTCGAGTGGCAATGGACTCAGGTTAT |  |  |
| MEOX2 | F  R | CGCTGTTTAAACCCAGAGGTGTTGGTTGTGTG | 422 |  |
|  |  | CTGTCTCGAGGCTGGTTCTGTTTGTCATCG |  |  |
| MARCH2 | F  R | CGCTGTTTAAACAGCCGATTCTGTGATTCCTG | 324 | Gradient PCR  (52.3-9.6) |
|  |  | CTGTCTCGAGGGGCTCCTTTTATTCATTCG |  |  |
| DOCR1 | F  R | CGCTGTTTAAACCGACTCCACCTCAGCTTCTGG | 199 | Touchdown |
|  |  | CTGTCTCGAGCCTCGCTGCTAACTCTTTCG |  |  |

Additional file 1: Table S1. List of primers used for validation of the miR-130b target genes.
