# Supplementary material for: MicroRNA-130b is involved in bovine granulosa and cumulus cells function, oocyte maturation and blastocyst formation
Source: J Ovarian Res. 2017 Jun 19;10:37. doi: 10.1186/s13048-017-0336-1 (PMC5477299; doi:10.1186/s13048-017-0336-1)
Supplement: Supplementary file 2 — List of primers used for quantification of the mRNA abundance level using qPCR. (DOCX 16 kb) [file 13048_2017_336_MOESM2_ESM.docx]

| Gene symbol | Accession. No |  | 5´ to 3´ | Annealing temperature (°C) |
| --- | --- | --- | --- | --- |
| MSK1 (RPS6KA5) | NM_001192023.1 | Forward | CTTGATTCTAATGGCCACGTGA | 53 |
|  |  | Reverse | CATCAACAGTGAACGGAGATGC |  |
| SMAD5 | NM_001077107.2 | Forward | CCATCAGCCCAACAACACT | 52 |
|  |  | Reverse | AGGCAGGAGGAGGAGTATCA |  |
| GAPDH | NM_001034034 | Forward | AATGGAAAGGCCATCACCATC | 57 |
|  |  | Reverse | GTGGTTCACGCCCATCACA |  |
| H2A | NM_174809.2 | Forward | GCCGTATTCATCGACACCTGA | 55 |
|  |  | Reverse | CTCCACGAATAGCAAGTTGCAA |  |
| pmirGLO | | Forward | GTGGTGTTGTGTTCGTGGAC | 58 |
|  |  | Reverse | CTTTCGGGCTTTGTTAGCAG |  |
| M13 | | Forward | TTGTAAACGCGGCCAGT | 59 |
|  |  | Reverse | CAGGAAACAGCTATGACC |  |

Additional file 2: Table S2. List of primers used for quantification of the mRNA abundance level using qRT-PCR
