# Supplementary figures and images for: MicroRNA-130b is involved in bovine granulosa and cumulus cells function, oocyte maturation and blastocyst formation
Source: J Ovarian Res. 2017 Jun 19;10:37. doi: 10.1186/s13048-017-0336-1 (PMC5477299; doi:10.1186/s13048-017-0336-1)

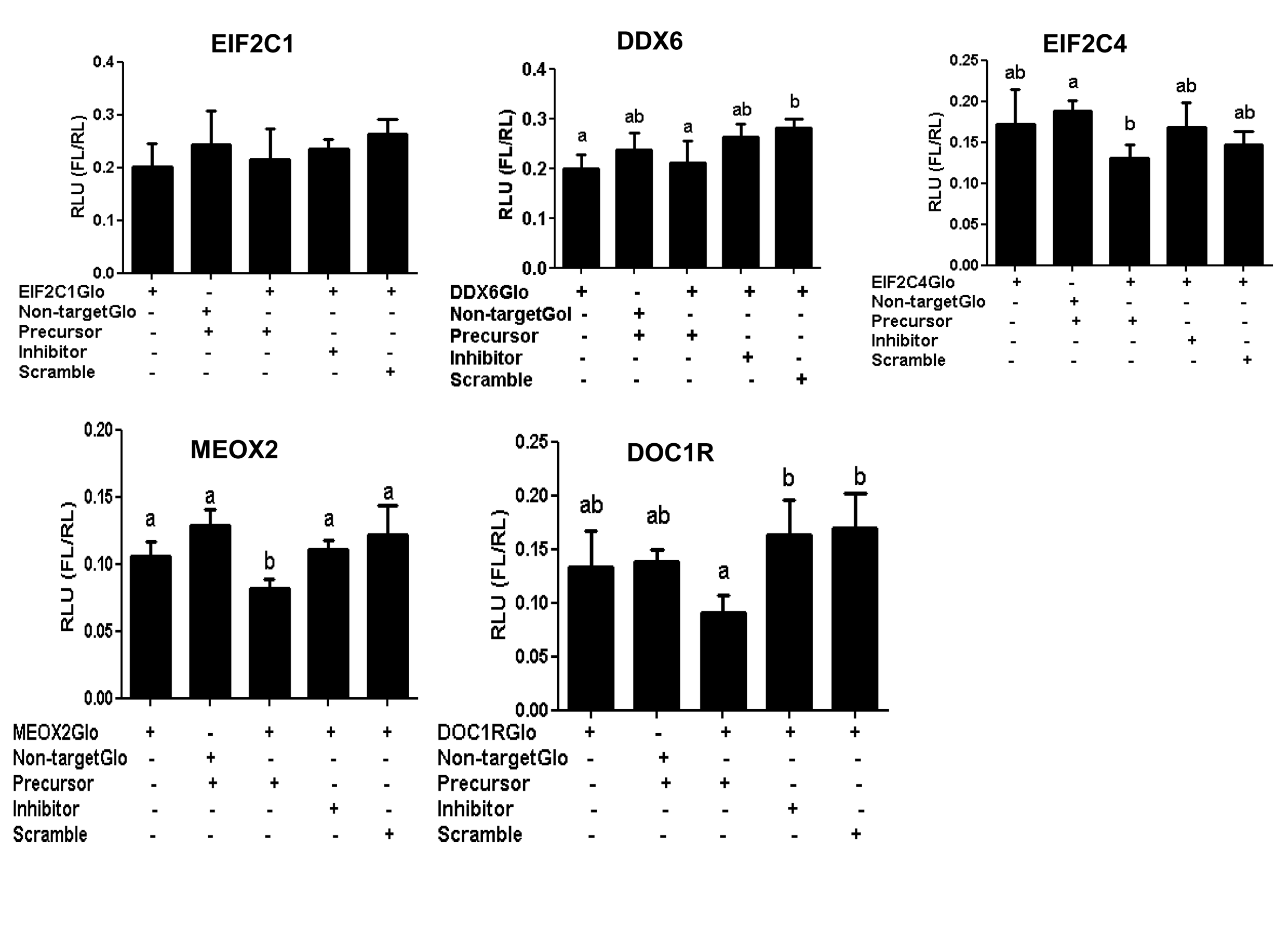

Supplement: Supplementary file 3 — The luciferase activity in cumulus cells co-transfected with and miR-130b precursor, miR-130b inhibitor, or scramble sequence with pmirGLO vector construct harboring the 3′ UTRs of EIF2C1, DDX2, EIF2C4, MEOX2 and DOC1R. (TIFF 1411 kb) [file 13048_2017_336_MOESM3_ESM.tif]
